# The Lived Experiences of Children Who Have Undergone Kidney Replacement Therapy and Their Families: Protocol for a Qualitative Systematic Review

**DOI:** 10.2196/77725

**Published:** 2026-01-12

**Authors:** Kenji Takao, Maki Fujitsuka, Shingo Ueki, Tomoyuki Sakai

**Affiliations:** 1 Department of Nursing, Faculty of Nursing, Osaka Dental University, 11-8 Kuzuha Hanazono-cho, Hirakata, 573-1121, Japan, 1 72864 ext 3658; 2 Department of Health Sciences, Graduate School of Medical Sciences, Kyushu University, Fukuoka, Japan; 3 Department of Pediatrics, Shiga University of Medical Science, Otsu, Shiga, Japan

**Keywords:** end-stage kidney disease in children, kidney replacement therapy, patient and family experience, qualitative research, quality of life

## Abstract

**Background:**

In many high-income countries, 5 to 10 children per million of the age-related population start kidney replacement therapy (KRT), which includes both dialysis (peritoneal dialysis and hemodialysis) and kidney transplantation (KT) for end-stage kidney disease. After peritoneal dialysis is introduced, or after KT, self-care at home typically becomes the main focus. Providing support for each developmental stage and transition period in the treatment process from dialysis to KT is an urgent issue.

**Objective:**

This review aims to synthesize the lived experiences of children undergoing KRT and their families. We will use identified themes to develop a self-care program aimed at solving the life tasks of children and their families.

**Methods:**

A search strategy will follow the Joanna Briggs Institute methodology and will be conducted in 3 steps: an initial limited search, a comprehensive database search, and a reference search of the included articles. MEDLINE (EBSCO), CINAHL Plus, and PsycINFO will be searched with no restriction on language or publication dates. The study selection, critical appraisal, data extraction, and data synthesis will be performed according to Joanna Briggs Institute guidelines for systematic reviews of qualitative research. Final synthesis will be assessed using the ConQual (confidence in the output of qualitative research synthesis) approach.

**Results:**

The review will include studies focusing on the experiences of children with KRT and their families. These experiences include physical, mental, and social issues, hygiene care, diet, fluid intake, medication, strict infection prevention, delays in growth and development, restrictions on social life, and a lack of social resources. This is an important issue because after starting dialysis, or after KT, many children are treated at home and experience these issues in their local communities and homes. As of May 2025, the authors have conducted 2 pilot searches to test and refine keywords of results with the help of the librarian and have identified 1003 studies for screening in MEDLINE. This systematic review is scheduled to be completed by April 2026.

**Conclusions:**

This systematic review synthesizes qualitative evidence regarding the daily life experiences of children and their families after initiating KRT, contributing to the development of a self-care program that enhances their quality of life.

## Introduction

### Background

End-stage kidney disease (ESKD) in children is a rare and irreversible condition that develops as a result of chronic kidney disease. Previous studies in many high-income countries have reported that approximately 5-10 children per million of the age-related population experience ESKD and undergo kidney replacement therapy (KRT) each year, although definitions and selection criteria vary from country to country [1-3]. The most common causes of ESKD in children are congenital anomalies of the kidney and urinary tract and hereditary nephropathy [4-6]. However, because ESKD is also associated with preterm birth and low birth weight, its incidence is increasing worldwide with recent advances in neonatal care [7]. Treatment for ESKD in children involves various KRT methods, such as hemodialysis (HD), peritoneal dialysis (PD), and kidney transplantation (KT), although treatment may be limited by financial, social, or religious constraints [8]. PD is considered the first choice for dialysis in children because it does not require painful vascular access, has less of an effect on circulation, and involves less strict dietary restrictions than HD. Additionally, it is easier for children to go to school or kindergarten while receiving home health care [9]. In most high-income countries, national registries for KT (including dialysis) have been implemented to describe the global incidence of pediatric KRT and to capture variations in treatment and outcomes [2]. As a result of advances in KRT, children and their families can now receive treatment not only in hospitals but also in their own communities. KRT cannot be maintained without being positioned within the lives of children and their families, including their growth and development, so self-care to improve quality of life (QOL) becomes a necessity.

Previous studies on QOL have reported that children on dialysis and children who have received a KT exhibit significantly impaired health-related QOL [10,11]. In addition, children with ESKD have been found to exhibit a significant decrease in health-related QOL at all stages [12]. In addition, KT has been found to improve QOL compared with dialysis, and children who have received KT exhibit better growth, neurocognitive development, academic performance, and QOL than children who have received dialysis [13]. KT represents an essential treatment for children in terms of physical and psychomotor development, and the benefits of successful KT are immense. According to the North America Pediatric Renal Transplant Cooperative Study of 2495 children under 18 years of age at the time of transplant, the rate of graft survival in the preemptive renal transplant (PRT) group was clearly superior to that in the non-PRT group [14]. Additionally, the estimated life expectancy of pediatric transplant patients is 30 to 40 years longer than that of pediatric dialysis patients [14]. The importance of early KT has also been demonstrated in terms of life prognosis [15]. However, there are also issues with donors and medical facilities in PRT, and children with ESKD often cannot avoid dialysis. Therefore, the transition to dialysis or kidney transplants is a continuous process that children and their families follow as KRT, and they face many physical, mental, and social difficulties in their daily lives, either for short periods of time or across developmental stages. For example, with regard to oral medication, infants and preschool children may find it difficult to take medication due to the bitter taste, discomfort, or shape of the medicine, and even if they do manage to take it, it may induce vomiting, which can lead to distress when they have to take it again. For school-age children and adolescents, the factors that hinder medication adherence are said to be at the individual, family, health care system, and community levels [16]. Difficulties in daily life not only affect children’s self-care but also the QOL of children and their families. It is necessary to consider ways to improve the issues in daily life in accordance with the developmental stage of the child.

We conducted a preliminary search of the following databases in April 2025: PROSPERO, MEDLINE (EBSCO), CINAHL Plus (EBSCOhost), and Joanna Briggs Institute (JBI) Evidence Synthesis. Few systematic reviews have examined KRT. Previous reviews can be divided into reviews focusing on children who have experienced PD/HD or their families [17,18] and those focusing on children who have experienced KT or their families [19]. However, children with ESKD often undergo repeated KRT throughout their lives. Thus, no previous systematic reviews have attempted to synthesize the qualitative evidence regarding the lived experiences of PD/HD and KT in children with ESKD and their families. Although substantial progress has been made in the development of KRT methods, the issues facing children and their families remain unclear. It is necessary to clarify these difficulties and to ensure that holistic medical care is provided for children and their families by the medical staff involved [20].

### Objectives

This systematic review will synthesize evidence regarding the life experiences of children undergoing KRT and their families and to clarify the life issues. We aim to develop a life-support system focused on the perspectives of children undergoing KRT and their families. The primary and secondary objectives of this study are as follows:

- The primary objective is the needs and dissatisfaction experienced by children and their families undergoing KRT.
- The secondary objective is the contextual and cultural factors influencing these family experiences, as well as the interactions between children and families.

### Review Questions

The review question for this study is “What needs and dissatisfaction are experienced by children and their families in daily life during KRT, including dialysis (PD/HD) and KT?” The subquestions are as follows:

- What are the contextual and cultural factors that influence the experiences of these children and families?
- What is the type of interaction between the children and their families?

## Methods

### Methodology and Protocol Registration

The proposed systematic review will be conducted in accordance with the JBI methodology for systematic reviews of qualitative evidence [21]. This protocol was registered in PROSPERO (CRD 42025644504).

### Eligibility Criteria

Eligible studies will include qualitative research designs (eg, phenomenology, grounded theory, ethnography, and qualitative descriptive studies) focusing on patient or family experiences after the initiation of KRT.

Quantitative studies, mixed method studies without extractable qualitative data, editorials, and commentaries will be excluded.

### Population

The proposed review will include studies of children who have undergone KRT for ESKD (glomerular filtration rate <15 ml/min/1.73 m^2^) when they were 18 years or younger and their families. Although no age limit at the time of study participation will be applied, it is likely that young adults (under 39 y) will be selected because the use of PD began in the 1980s, followed by the development of automated PD and continuous hemodiafiltration in the 1990s, as well as the advent of superior immunosuppressants [9]. We will include cases of families that meet the inclusion criteria even if their child is no longer alive when the study recruitment takes place. KRT includes 3 types of treatment: PD, HD, and KT. KT includes both living-donor and deceased-donor KT. Additionally, KT also includes PRT, in which a kidney is transplanted before dialysis is needed, as well as including dialysis after PRT and second transplants. Data extraction will be conducted independently by two reviewers using a predeveloped standardized form. Information will be extracted separately for each treatment modality—HD, PD, and KT. For each included study, the following population characteristics will be extracted (where available): age range or mean age of participants, sex distribution (male/female), underlying kidney disease (including proportions by diagnostic category, if reported), comorbidities or complications, and family relationship to the patient (eg, parent, child, caregiver).

These details will help contextualize the diversity of participant backgrounds and ensure comparability across studies.

In this review, “family” is defined as a person identified as the primary caregiver for the child or as holding a significant role within the household. Therefore, biological parents, siblings, and grandparents are included as family members. Nonbiological relatives such as adoptive parents, parents of children born through surrogacy, and stepparents are also included. During screening, both reviewers will independently confirm whether the study explicitly describes participants as family members or primary caregivers. Any ambiguity will be clarified by contacting the authors to clarify or consulting with a third reviewer (SU).

### Phenomena of Interest

This review will consider research that explores the lived experiences of children who have undergone KRT (ie, PD, HD, and KT) and their families. These experiences will include individuals’ demands and needs regarding KRT in daily life.

### Context

This review will consider studies that are conducted in any context of the children’s lives to reflect the diverse voices of children and families with KRT. This includes the community, school, family settings, leisure activities, and visits to hospitals. Moreover, this review will not be restricted to any country or region.

### Types of Studies

We will consider all studies in which qualitative data were collected, including, but not limited to, designs such as phenomenology, grounded theory, ethnography, action research, discourse analysis, and qualitative descriptions. Qualitative components of mixed methods studies that contain qualitative data will also be included if the qualitative results are reported separately. We will review academic papers, theses and dissertations, and conference papers. Moreover, the research will only include studies in which informed consent was obtained from the parents and informed assent was obtained from the children to ensure thorough consideration of ethical aspects in our review because the research involves minors.

### Search Strategy

The search strategy will aim to locate both published and unpublished studies. An initial limited search of MEDLINE (EBSCO) and CINAHL was undertaken to identify articles on the topic. The text words contained in the titles and abstracts of relevant articles and the index terms used to describe the articles were used to develop a full search strategy (Table 1). The search strategy, including all identified keywords and index terms, will be adapted for each included information source. The reference list of all studies selected for critical appraisal will be screened for additional studies. There will be no restrictions in terms of language or year of publication. For studies published in languages other than English and Japanese, we will use translation software. The databases to be searched will include MEDLINE (EBSCO), CINAHL, PsycINFO, and Igaku Chuo Zasshi. The unpublished studies will be searched using Mednar. Gray literature will be searched using ProQuest Dissertations and Theses and Google Scholar.

**Table 1.** Search conducted on MEDLINE (EBSCO) on April 23, 2025.

Search	Query	Records retrieved, n
1^a^	MH^b^ “Renal Replacement Therapy+” OR MH “Renal Dialysis+” OR MH “Peritoneal Dialysis+” OR (MH “Kidney Transplantation” OR MH “Organ Transplantation+")	380,767
2^a^	MH “Child+” OR MH “Infant+” OR MH “Adolescent” OR MH “Minors+” OR MH “Family+” OR MH “Parents+” OR MH “Caregivers”	4,346,908
3^c^	MH “Adverse Childhood Experiences” OR MH “health services needs and demand” OR MH Stress, Psychological OR MH Quality of Life OR MM^d^ “Patient Preference” OR MH “Decision Making+” OR MH “Patient-Centered Care+”	756,711
4^c^	XB^e^ (experience* OR demand* OR need* OR stress* OR feel* OR anxiety* OR burden* OR preference* OR dissatisf* OR life OR live*)	7,886,771
5	3 OR 4	8,137,015
6^f^	(MH “Interviews as Topic” OR MH “Interview”) OR MH “Focus Groups” OR MH “Qualitative Research+” OR MH “Grounded Theory” OR MH “Anthropology, Cultural+”	390,534
7^f^	XB (qualitative* OR Narrative OR ethno* OR Phenomenology OR “action research” OR “content analysis” OR “thematic analysis” OR interview* OR “mixed method*”)	1,158,783
8	6 OR 7	1,320,861
9	1 AND 2 AND 5 AND 8	1003

a 1 and 2: Population.

b MH: MeSH heading.

c 3 and 4: Phenomena of interest.

d MM: major MeSH heading

e XB: exploded MeSH heading.

f 6 and 7: Methods and data collection approaches related to qualitative research.

### Study Selection

Following the search, all identified citations will be collated and uploaded into EndNote 21 (Clarivate Analytics), and duplicates will be removed. A pilot screening involving 30 studies will then be conducted by 2 reviewers (KT and MF) to independently review the titles and abstracts. We will continue to perform pilot tests and engage in discussion until agreement is reached. The titles and abstracts will then be screened against the review inclusion criteria. Potentially relevant studies will be retrieved in full and their citation details imported into the JBI System for the Unified Management, Assessment, and Review of Information (JBI SUMARI; JBI) [22].

The full text of selected citations will be assessed in detail against the inclusion criteria by 2 independent reviewers. Reasons for exclusion of full-text studies that do not meet the inclusion criteria will be recorded and reported in the systematic review. Any disagreements that arise between the reviewers at each stage of the study selection process will be resolved through discussion or with a third reviewer (SU).

### Assessment of Methodological Quality

Eligible studies will be critically appraised by 2 independent reviewers (KT and MF) for methodological quality using the standard JBI critical appraisal checklist [21] for qualitative research. Authors of studies will be contacted to request missing or additional data for clarification, where required. The evaluation content of the checklist includes congruity between the stated philosophical perspective and the research methodology; congruity between the research methodology and the research question or objectives; congruity between the research methodology and the methods used to collect data; congruity between the research methodology and the representation and analysis of data; congruity between the research methodology and the interpretation of results; a statement locating the researcher culturally or theoretically; the influence of the researcher on the research, and vice versa, addressed; are participants, and their voices, adequately represented; evidence of ethical approval by an appropriate body; and the conclusions drawn in the research report flow from the analysis, or interpretation, of the data. All items were evaluated by using “yes,” “no,” “unclear,” and “not applicable” to appraisal. The appraisal items are listed in Table 2. Any disagreements that arise between the reviewers will be resolved through discussion or with a third reviewer (SU). The results of critical appraisal will be reported in narrative form and in formats. Following critical appraisal, only studies rated “no” for Q8 (Are participants, and their voices, adequately represented?) will be excluded. This item is treated as a methodological requirement specific to JBI meta-aggregation because findings must be supported by participant quotations (illustrations). When participant voices are not adequately represented, the necessary illustrations for extraction and credibility assessment are unavailable, making synthesis technically impossible. Therefore, excluding studies with a “no” rating on Q8 is not a quality-based threshold but a requirement for data extractability. For all other items on the checklist, studies rated “no” or “unclear” will be included as these limitations do not prevent extraction of findings and illustrations. If there is insufficient information to assess methodological quality, it will be rated as “unclear” without contacting the study authors.

**Table 2.** Joanna Briggs Institute quality appraisal tool.

	Yes	No	Unclear	N/A^a^
1. Is there congruity between the stated philosophical perspective and the research methodology?	□	□	□	□
2. Is there congruity between the research methodology and the research question or objectives?	□	□	□	□
3. Is there congruity between the research methodology and the methods used to collect data?	□	□	□	□
4. Is there congruity between the research methodology and the representation and analysis of data?	□	□	□	□
5. Is there congruity between the research methodology and the interpretation of results?	□	□	□	□
6. Is there a statement locating the researcher culturally or theoretically?	□	□	□	□
7. Is the influence of the researcher on the research, and vice versa, addressed?	□	□	□	□
8. Are participants, and their voices, adequately represented?	□	□	□	□
9. Is the research ethical according to current criteria or, for recent studies, and is there evidence of ethical approval by an appropriate body?	□	□	□	□
10. Do the conclusions drawn in the research report flow from the analysis, or interpretation, of the data?	□	□	□	□
Overall appraisal: Include □ Exclude □ Seek further info □				
Comments (Including reason for exclusion)				

a Not Applicable.

### Data Extraction

Data will be extracted from studies included in the review by 2 independent reviewers (KT and MF) using the standardized JBI data extraction tool in JBI SUMARI [22]. The extracted data will include specific details about the populations, context, culture, geographical location, study methods, and the phenomena of interest relevant to the review objective. In this review, context refers to family composition, the setting where treatment occurs (hospital, home, etc), health care and insurance systems, and schooling. Culture refers to lifestyle habits such as attitudes toward treatment (views on dialysis and transplantation), family roles, and dietary customs. Focusing on and exploring the daily lives of children and their families, their findings and illustrations will be extracted and assigned a level of credibility. When required, authors of specific studies will be contacted to request missing or additional data.

### Data Synthesis

Qualitative research findings will, where possible, be pooled using JBI SUMARI with the meta-aggregation approach. This will involve the aggregation or synthesis of findings to generate a set of statements that represent that aggregation, through assembling the findings and categorizing these findings on the basis of similarity in meaning. These categories will then be subjected to a synthesis in order to produce a single comprehensive set of synthesized findings that can be used as a basis for evidence-based practice. Where textual pooling is not possible, the findings will be presented in narrative form. Only unequivocal and credible findings will be included in the synthesis. The terms “unequivocal” and “credible” will follow the JBI’s explanation:

- Unequivocal relates to evidence beyond reasonable doubt, which may include conclusions that are matter of fact directly reported/observed and not open to challenge.
- Credible relates to those conclusions that are, albeit interpretations, plausible in light of the textual data and theoretical framework. As the conclusions are interpretive, they can be challenged.

Only findings classified as unequivocal or credible will be included in the synthesis; unsupported statements will be excluded.

#### Assessing Confidence in the Findings

The methodological quality of included studies will be appraised using the JBI Critical Appraisal Checklist for Qualitative Research (Table 2).

The ConQual (confidence in the output of qualitative research synthesis) approach will be used to establish the confidence level in each synthesized finding [23] (Textbox 1).

Each finding will receive an initial high score that is downgraded based on 2 domains: dependability (reflecting methodological rigor) and credibility (reflecting the strength of evidence support).

A summary table will present the final ConQual scores for all synthesized findings.

The summary of findings will include the major elements of the review and detail how the ConQual score has been developed and the title, population, phenomena of interest, and context for the specific review.

Textbox 1. **ConQual (confidence in the output of qualitative research synthesis) summary of findings**

Review title: The lived experiences of children who have undergone kidney replacement therapy (KRT) and their families: a qualitative systematic review

Population: children who have undergone KRT for end-stage kidney disease when they were 18 years old or younger and their families.

Phenomena of interest: the exposition of lived experiences of children who have undergone KRT and their families.

Context: the lived experiences of children who have undergone KRT and their families.

Summary of findings will include:

- Synthesized finding
- Type of research
- Dependability
- Credibility
- ConQual score

## Results

**Figure 1.**
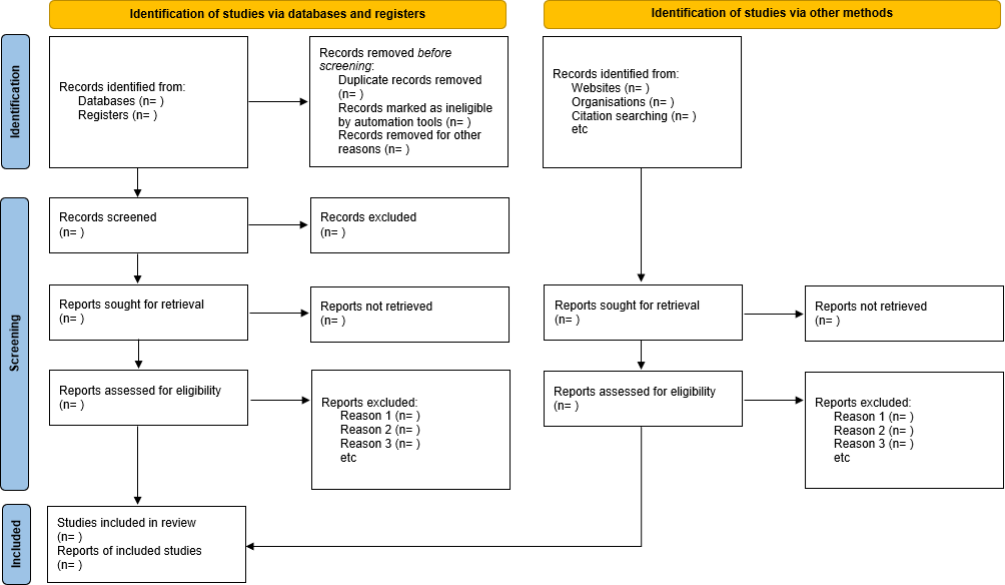
PRISMA (Preferred Reporting Items for Systematic Reviews and Meta-Analyses) 2020 flow diagram for new systematic reviews which included searches of databases, registers, and other sources. If feasible to do so, we will report the number of records identified from each database or register searched (rather than the total number across all databases/registers). If automation tools were used, we will indicate how many records were excluded by a human and how many were excluded by automation tools. Source: Page et al [24].

The results will report on all studies concerning children and families’ experiences with daily life focusing on KRT, including cleanliness care, diet, fluid intake, medication, strict infection prevention, delay in growth and development, restriction of social life, and insufficient social resources. The results of the search will be reported in full in the final systematic review and presented in a PRISMA (Preferred Reporting Items for Systematic Reviews and Meta-Analyses) flow diagram (Figure 1) [24].

As of May 2025, the authors have conducted 2 pilot searches to test and refine keywords of results with the help of the librarian. We have identified 1003 studies for screening in MEDLINE. We will apply the number of records identified, screened, excluded, and included at each stage in Figure 1, after the full search and study selection are performed. This qualitative synthesis will determine the current studies exploring the lived experiences of children undergoing KRT and their families. The results will presumably report on 3 treatment types such as PD, HD, and KT.

We will summarize our findings in a table and a conceptual diagram and discuss them in a descriptive and narrative review form. We will discuss the implications for future research, policies, and practices. Our findings will be presented at a relevant conference and submitted in a peer-reviewed journal. The review team is currently engaged in data extraction, and this systematic review is expected to be completed by April 2026.

## Discussion

### Principal Findings

Children with ESKD must continue to undergo some form of KRT throughout their lives. To our knowledge, there is no other qualitative synthesis investigating the experiences of children undergoing KRT (particularly from dialysis to KT) and their families. This qualitative synthesis will be unique in identifying issues for seamless support for children and their families. Globally, there is a need for health care that emphasizes the perspectives of patients and their families. It is essential to investigate the real voices of patients and their families. By conducting a systematic review from the perspective of patients and their families, it will be possible to bridge the gap between the care provided by health care professionals and the care sought by patients and their families.

### Strengths and Limitations of This Study

The qualitative systematic review will evaluate the lived experiences from the perspectives of both children undergoing KRT and their families and may reveal comprehensive demands and mutually beneficial opportunities for qualitative improvement for the whole family. Dialysis and kidney transplants are integrated into a comprehensive process of living experience as KRT. This study included the literature from multidisciplinary databases, gray literature, and all languages to maximize data diversity and comprehensiveness. Human errors are minimized by having two independent researchers carry out literature selection, quality assessment, and data extraction according to the JBI methodology. Meta-integration will be performed to gain a comprehensive understanding of the topic and identify gaps for further research.

### Conclusions

This systematic review protocol aims to comprehensively synthesize qualitative research findings regarding the daily life experiences—such as difficulties, needs, and dissatisfactions—faced by children and their families after initiating KRT.

We will use identified themes to develop a self-care program aimed at solving the life tasks of children and their families. This research will contribute to the realization of a society in which the QOL of children and their families is ensured to the greatest extent possible.

## Abbreviations

ConQual: confidence in the output of qualitative research synthesis
ESKD: end-stage kidney disease
HD: hemodialysis
JBI: Joanna Briggs Institute
JBI SUMARI: JBI System for the Unified Management, Assessment, and Review of Information
KRT: kidney replacement therapy
KT: kidney transplantation
PD: peritoneal dialysis
PRISMA: Preferred Reporting Items for Systematic Reviews and Meta-Analyses
PRT: preemptive renal transplant
QOL: quality of life

## References

[1] Harambat J, van Stralen KJ, Kim JJ, Tizard EJ (2012). Epidemiology of chronic kidney disease in children. Pediatr Nephrol.

[2] Ploos van Amstel S, Noordzij M, Warady BA (2018). Renal replacement therapy for children throughout the world: the need for a global registry. Pediatr Nephrol.

[3] Maurer E, Neuhaus TJ, Weitz M, Kuehni CE, Laube GF (2020). Paediatric end-stage renal disease and renal replacement therapy in Switzerland: survival and treatment trends over four decades. Swiss Med Wkly.

[4] Mong Hiep TT, Ismaili K, Collart F (2010). Clinical characteristics and outcomes of children with stage 3-5 chronic kidney disease. Pediatr Nephrol.

[5] Ardissino G, Daccò V, Testa S (2003). Epidemiology of chronic renal failure in children: data from the ItalKid project. Pediatrics.

[6] Ishikura K, Uemura O, Ito S (2013). Pre-dialysis chronic kidney disease in children: results of a nationwide survey in Japan. Nephrol Dial Transplant.

[7] Sanderson KR, Shih WV, Warady BA, Claes DJ (2024). Severe fetal CAKUT (Congenital Anomalies of the Kidneys and Urinary Tract), prenatal consultations, and initiation of neonatal dialysis. Am J Perinatol.

[8] Lalji R, Francis A, Wong G (2020). Disparities in end-stage kidney disease care for children: a global survey. Kidney Int.

[9] Hattori M (2009). The history and current state of the treatment of end-stage chronic renal failure in children: from the 53rd Japanese Society for Dialysis Therapy Educational Lecture. J Jpn Soc Dial Ther.

[10] Splinter A, Tjaden LA, Haverman L (2018). Children on dialysis as well as renal transplanted children report severely impaired health-related quality of life. Qual Life Res.

[11] Doshi K, Raina R, Ng KH (2024). Health-related quality of life for pediatric patients with end-stage kidney disease: a systematic review and meta-analysis of the Pediatric Quality of Life Inventory (PedsQL). Hemodial Int.

[12] Francis A, Didsbury MS, van Zwieten A (2019). Quality of life of children and adolescents with chronic kidney disease: a cross-sectional study. Arch Dis Child.

[13] Francis A, Johnson DW, Melk A (2020). Survival after kidney transplantation during childhood and adolescence. Clin J Am Soc Nephrol.

[14] Vats AN, Donaldson L, Fine RN, Chavers BM (2000). Pretransplant dialysis status and outcome of renal transplantation in North American children: a NAPRTCS Study12. Transplantation.

[15] McDonald SP, Craig JC, Australian and New Zealand Paediatric Nephrology Association (2004). Long-term survival of children with end-stage renal disease. N Engl J Med.

[16] Varnell CD, Rich KL, Nichols M (2017). Assessing barriers to adherence in routine clinical care for pediatric kidney transplant patients. Pediatr Transplant.

[17] Tjaden L, Tong A, Henning P, Groothoff J, Craig JC (2012). Children’s experiences of dialysis: a systematic review of qualitative studies. Arch Dis Child.

[18] Tong A, Lowe A, Sainsbury P, Craig JC (2008). Experiences of parents who have children with chronic kidney disease: a systematic review of qualitative studies. Pediatrics.

[19] Araújo NSS, Pereira RRF, Fram D, Hino P, Longo MCB, Taminato M (2018). Quality of life in children with kidney transplant: systematic review. Rev Bras Enferm.

[20] Gotoh Y, Nagai T, Yamakawa S, Uemura O (2008). The present conditions in the pediatric renal transplant and problems: based on a pediatric renal transplant patients of 55 this hospital. Jpn J Pediatr Nephrol.

[21] Porritt K, Evans C, Bennett C, Aromataris E, Lockwood C, Porritt K, Pilla B, Jordan Z (2024). JBI Manual for Evidence Synthesis.

[22] Munn Z, Aromataris E, Tufanaru C (2019). The development of software to support multiple systematic review types: the Joanna Briggs Institute System for the Unified Management, Assessment and Review of Information (JBI SUMARI). Int J Evid Based Healthc.

[23] Munn Z, Porritt K, Lockwood C, Aromataris E, Pearson A (2014). Establishing confidence in the output of qualitative research synthesis: the ConQual approach. BMC Med Res Methodol.

[24] Page MJ, McKenzie JE, Bossuyt PM (2021). The PRISMA 2020 statement: an updated guideline for reporting systematic reviews. BMJ.

